# Efficiency of 7.2% hypertonic saline hydroxyethyl starch 200/0.5 versus mannitol 15% in the treatment of increased intracranial pressure in neurosurgical patients – a randomized clinical trial [ISRCTN62699180]

**DOI:** 10.1186/cc3767

**Published:** 2005-08-09

**Authors:** Lilit Harutjunyan, Carsten Holz, Andreas Rieger, Matthias Menzel, Stefan Grond, Jens Soukup

**Affiliations:** 1 Anaesthesiologist, Department of Anesthesia and Critical Care, Martin-Luther-University Halle-Wittenberg, Halle, Germany; 2 Neurosurgeon, Department of Neurosurgery, Martin-Luther-University Halle-Wittenberg, Halle, Germany; 3 Head, Department of Anesthesia and Critical Care, Klinikum Wolfsburg, Wolfsburg, Germany; 4 Professor of Anesthesiology and Pain Therapy, Department of Anesthesia and Critical Care, Martin-Luther-University Halle-Wittenberg, Halle, Germany; 5 Anaesthesiologist and Intensivist, Department of Anesthesia and Critical Care, Martin-Luther-University Halle-Wittenberg, Halle, Germany

## Abstract

**Introduction:**

This prospective randomized clinical study investigated the efficacy and safety of 7.2% hypertonic saline hydroxyethyl starch 200/0.5 (7.2% NaCl/HES 200/0.5) in comparison with 15% mannitol in the treatment of increased intracranial pressure (ICP).

**Methods:**

Forty neurosurgical patients at risk of increased ICP were randomized to receive either 7.2% NaCl/HES 200/0.5 or 15% mannitol at a defined infusion rate, which was stopped when ICP was < 15 mmHg.

**Results:**

Of the 40 patients, 17 patients received 7.2% NaCl/HES 200/0.5 and 15 received mannitol 15%. In eight patients, ICP did not exceed 20 mmHg so treatment was not necessary. Both drugs decreased ICP below 15 mmHg (*p *< 0.0001); 7.2% NaCl/HES 200/0.5 within 6.0 (1.2–15.0) min (all results are presented as median (minimum-maximum range)) and mannitol within 8.7 (4.2–19.9) min (*p *< 0.0002). 7.2% NaCl/HES 200/0.5 caused a greater decrease in ICP than mannitol (57% vs 48%; *p *< 0.01). The cerebral perfusion pressure was increased from 60 (39–78) mmHg to 72 (54–85) mmHg by infusion with 7.2% NaCl/HES 200/0.5 (*p *< 0.0001) and from 61 (47–71) mmHg to 70 (50–79) mmHg with mannitol (*p *< 0.0001). The mean arterial pressure was increased by 3.7% during the infusion of 7.2% NaCl/HES 200/0.5 but was not altered by mannitol. There were no clinically relevant effects on electrolyte concentrations and osmolarity in the blood. The mean effective dose to achieve an ICP below 15 mmHg was 1.4 (0.3–3.1) ml/kg for 7.2% NaCl/HES 200/0.5 and 1.8 (0.45–6.5) ml/kg for mannitol (*p *< 0.05).

**Conclusion:**

7.2% NaCl/HES 200/0.5 is more effective than mannitol 15% in the treatment of increased ICP. A dose of 1.4 ml/kg of 7.2% NaCl/HES 200/0.5 can be recommended as effective and safe. The advantage of 7.2% NaCl/HES 200/0.5 might be explained by local osmotic effects, because there were no clinically relevant differences in hemodynamic clinical chemistry parameters.

## Introduction

The development or presence of secondary brain injury in patients with intracranial pathology has been associated with increased morbidity and mortality. An increase in intracranial pressure (ICP) accompanied by a low cerebral perfusion pressure (CPP) should therefore be avoided in these patients. Several clinical studies have demonstrated that outcome is improved by adequate pharmacological or neurosurgical treatment optimizing ICP [1-3]. According to established treatment guidelines, an ICP >20 mmHg and a CPP <60 mmHg are considered critical [4-8]. Early recognition of such critical episodes by multimodal neuromonitoring, and selection of an effective and safe drug for treatment are essential for neuroprotection.

Osmotherapy has been used since the early 20^th ^century to treat increased ICP. The physiological basis and concept of osmotherapy was first published in 1919 [9]. Intravenous infusion of mannitol is considered to be the 'gold standard' for the treatment of increased ICP. Barbiturates and TRIS buffer are still used as alternative treatments, although their use in clinical practice is limited by cardiovascular and metabolic side effects [10-13]. In addition, experimental and clinical evidence has shown that 'small volume resuscitation' has a positive effect in the treatment of increased ICP in trauma patients [14-16].

Experimentally, intravenous application of hypertonic saline increases global cerebral perfusion as well as the right-shifted oxygen dissociation curve, both with consecutive improvement of oxygen delivery. At the same time, an increase of cerebral compliance and decrease in ICP occur by decrease of the brain edema [17].

Although several experimental and clinical studies have investigated the effects of hypertonic saline or mannitol on ICP, only a few studies comparing these drugs in neurosurgical patients have been published [18-22]. Furthermore, there are no clinical data available for recommendation of an 'effective dose' of hypertonic saline in clinical practice.

The purpose of this study was to compare the efficacy and safety of 7.2% NaCl/HES 200/0.5 and mannitol 15% in neurosurgical patients with increased ICP. This study focuses on the effects of both drugs on ICP, CPP, mean arterial pressure (MAP), hematocrit, serum sodium and osmolarity. Furthermore, we attempted to recommend an effective dose for the application of hypertonic saline.

## Methods

After approval by the local ethics committee and written informed consent being obtained from the patients' legal relatives, neurosurgical patients with severe neuronal damage (e.g. cerebral trauma, spontaneous intracerebral bleeding or subarachnoidal bleeding) were enrolled in this prospective randomized study. The patients were randomized to receive either 7.2% NaCl/HES 200/0.5 (HyperHAES^®^, Fresenius Kabi Deutschland GmbH, Bad Homburg) or mannitol (Osmofundin^® ^15%-N, B. Braun Melsungen AG, Melsungen, Germany), to treat increased ICP.

Inclusion criteria were: age >18 years, severe brain damage (Glasgow Coma Score <8) with cerebral edema – visualized by CT scan and continuous monitoring of ICP. Exclusion criteria were: elevated ICP due to space-occupying lesions with indication for neurosurgical intervention (e.g. bleeding, hydrocephalus), severe renal failure, metabolic disorders, initial serum sodium >150 mmol/l and initial serum osmolarity >320 mosm/kg.

### Standard treatment protocol

All patients were intubated and received pressure-controlled mechanical ventilation (Bilevel Positive Airway Pressure (BiPAP), etCO_2 _4.2–4.8 kPa, FiO_2 _0.3–1.0). Care was taken to keep the arterial partial oxygen pressure above 15 kPa, the hemoglobin concentration above 5.5 mmol/l and the CPP above 70 mmHg. If necessary, blood pressure was supported with vasopressor therapy. Blood glucose was adjusted to values between 6–8 mmol/l by continuous application of human insulin. Patients' core temperature was measured via the bladder, with a target temperature of 36.0–37.0°C. If the core temperature exceeded 37.0°C, external cooling blankets were used to cool the patient, otherwise patients were covered either with an additional blanket or with an active heating blanket (Bair Hugger; Augustine Medical, Eden Prairie, MN, USA).

Analgosedation and continuous patient monitoring were managed according to the standards of the Department of Anesthesiology and Critical Care at the Martin-Luther-University Halle-Wittenberg, Germany. Analgosedation at days 1–4 was performed using propofol and sufentanil or remifentanil. Thereafter, midazolam and sufentanil were administered. The standard monitoring included electrocardiogram, invasive arterial blood pressure, central venous pressure, peripheral oxygen saturation (SpO_2_) and intraparenchymal ICP measurement (Codman Microsensor ICP Monitoring System; Codman & Shurtleff Inc, Raynham, MA, USA).

An increase in ICP was treated first by deepening the sedation and analgesia by titrating the medication and adjusting to adequate ventilator settings. If ICP exceeded the 20 mmHg threshold for more than 5 min, the study medication (mannitol or 7.2% NaCl/HES 200/0.5 (herein referred to as '7.2% hypertonic saline' or 'hypertonic saline') was infused via the central venous line using an automated infusion system at a defined infusion rate. The infusion was stopped when ICP was reduced to <15 mmHg, defined as the treatment goal. However, in the case of sustained ICP problems (ICP >15 mmHg or CPP <70 mmHg) after these measures, bolus applications of thiopentone (maximum single bolus: 5 mg/kg) were allowed. In these patients, the possibility of a space-occupying lesion was excluded by CT scan.

### Data acquisition and statistical analysis

Mean arterial blood pressure, heart rate, SpO_2_, ICP and calculated CPP were continuously measured. Analysis of these parameters was performed at the following time points: initiation of infusion; after termination of infusion (ICP <15 mmHg achieved); 10 min after terminating infusion; 30 min after terminating infusion; and 60 min after terminating infusion. Serum sodium level and hematocrit were measured every 4 h and the serum osmolarity every 12 h. The values taken before the therapy, as well as the maximum values subsequently achieved, were analyzed. Individual outcomes were assessed at the end of stay in the intensive care unit (ICU) using the differentiation between survivors and non-survivors.

The random code for group assignment was generated by computer. The software package Stat View 4.0 (Abacus Concepts Inc, Berkeley, CA, USA) was used for all statistical calculations. All demographic data are presented as mean ± SD. The clinical values in both groups were not normally distributed. Results are presented as median (minimum-maximum range). Groups were compared using the non-parametric Mann-Whitney U-Test and the Wilcoxon Signed Rank was employed to analyze the effect of the medication used within each group; *p *< 0.05 was regarded as statistically significant and computed significance levels are given.

## Results

A total of 40 neurosurgical patients were recruited according to the inclusion criteria and randomized to receive either 7.2% NaCl/HES 200/0.5 (*n *= 17) or mannitol 15% (*n *= 15) to treat increased ICP. Only 32 patients were evaluated since in eight patients, ICP did not exceed 20 mmHg, therefore no study medication was administered.

Demographic data of all analyzed patients are summarized in Table 1. There were no significant differences between the two groups. No relevant clinical characteristics were revealed in the eight patients not undergoing osmotic therapy.

Analgosedation was started in all patients using our standard protocol. In four patients in the 7.2% hypertonic saline group and five patients in the mannitol group, propofol was substituted by thiopental because of sustained ICP problems.

### Heart rate and blood pressure

The average baseline heart rate was 78 (58–95) bpm in the mannitol and 76 (52–92) bpm in the hypertonic saline group (*p *= NS). The infusion of study medication produced no clinically relevant changes in heart rate and no arrhythmias.

**Figure 1:**
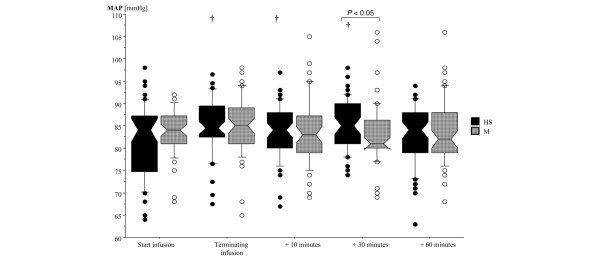
Box-and-whisker plots of the MAP. Data are plotted for the first hour after administration of 7.2% NaCl/HES 200/0.5 (HS) or mannitol 15% (M). In patients receiving 7.2% NaCl/HES 200/0.5, the MAP change was statistically significant compared with the value at the start of treatment († *p *< 0.05). The changes with mannitol were not statistically significant within the group, but significant after 30 min to HS (*p < 0.05). MAP, mean arterial pressure.

The initial MAP was 84 (68–92) mmHg in the mannitol group and 82 (64–98) mmHg in the hypertonic saline group (*p *= NS). Maximal changes could be analyzed in the mannitol group after 10 min (83 (69–105) mmHg) and in patients receiving hypertonic saline after 30 min (85 (74–98) mmHg) (Fig. 1, Table 2).

The individual maximum increase of MAP during the observation time after infusion of mannitol was 5.8% to 88 (72–106) mmHg and after infusion of hypertonic saline was 7.6% to 85 (74–98) mmHg. The time of the maximal increase was individual for each patient as well.

### ICP and CPP

**Figure 2:**
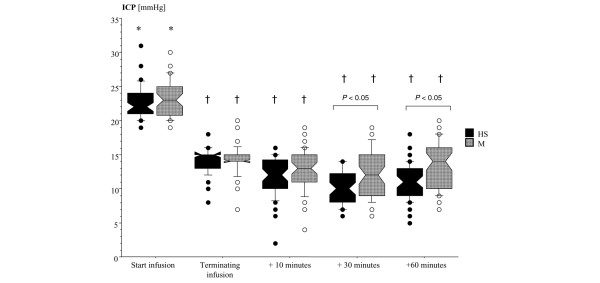
Box-and-whisker plots of the ICP. Data are plotted for the first hour after intravenous administration of 7.2% NaCl/HES 200/0.5 (HS) or mannitol (M). The ICP decreases after injection of the respective test substance significantly in comparison with the baseline value at the start of treatment († *p *< 0.0001). After 30 min and 60 min, a statistically significant difference was seen between the two treatment regimes (*p *< 0.05) ICP, intracranial pressure.

Prior to administration of the study medication, the mean ICP was 23 (19–30) mmHg in the mannitol group and 22 (19–31) mmHg in the hypertonic saline group (*p *= NS). After infusion with mannitol, the ICP decreased to 14 (7–20) mmHg and after infusion with hypertonic saline it decreased to 15 (8–18) mmHg (*p *< 0.0001). This effect was achieved within 8.7 (4.2–19.9) min by mannitol and 6.0 (1.2–15.0) min by hypertonic saline (*p *< 0.0002) and maintained over the 1 h observation period. The lowest ICP was 12 (6–19) mmHg in the mannitol and 10 (6–14) mmHg in the hypertonic saline group (*p *< 0.05), observed 30 min after the end of infusion. Thus, the maximum decrease in ICP produced by hypertonic saline was 57% and that of mannitol 48%. Sixty minutes after the end of infusion, the ICP in the hypertonic saline group was still lower than that of the mannitol group (11 (5–18) mmHg; vs 14 (7–20) mmHg; *p *< 0.005) (Fig. 2, Table 2).

**Figure 3:**
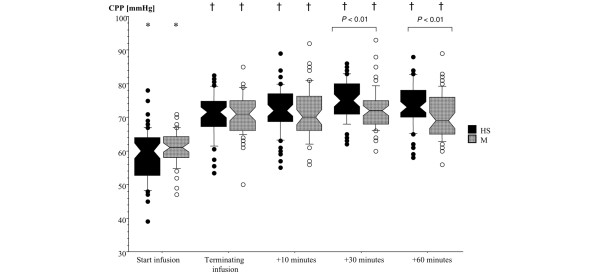
Box-and-whisker plots of the mean CPP. Data are plotted within the first hour after administration of 7.2% NaCl/HES 200/0.5 (HS) or mannitol (M). The CPP increases significantly compared with the start of treatment († *p *< 0.0001). After 30 min and 60 min, a statistically significant difference was seen between the two treatment regimes (*p *< 0.01). CPP, cerebral perfusion pressure.

Prior to administration of study medication, the mean CPP was 61 (47–71) mmHg in the mannitol and 60 (39–78) mmHg in the hypertonic saline group (*p *= NS; Fig. 3). At the end of infusion, a significant increase of CPP to 70 (50–79) mmHg after mannitol infusion (*p *< 0.0001) and 72 (54–85) mmHg after hypertonic saline infusion (*p *< 0.0001) occurred. This improvement was maintained during the whole study period. The maximal increase in CPP occurred in both groups after 30 min (mannitol +18%; hypertonic saline +27%; *p *< 0.05). CPP was significantly higher in the hypertonic saline group (*p *< 0.01, Fig. 3, Table 2) 30 and 60 min after the end of infusion.

The 15 patients in the mannitol group had a total of 53 episodes of increased ICP exceeding 20 mmHg requiring infusion of study medication (3.5 treatments/patient). For 49 of these episodes (92.5%), infusion of mannitol was effective and reduced ICP to <15 mmHg within 8.7 (4.2–19.9) min. For one episode, mannitol produced a delayed effect, appearing 20 min after application of a total of 235 ml mannitol (2.6 ml/kg). In three episodes, however, ICP could not be reduced below 15 mmHg by an infusion of up to 2.1 ml/kg of mannitol. In two of these patients, thiopental was given intravenously at up to 3 mg/kg and in one patient a unilateral decompressive craniectomy was performed.

In the 17 patients in the hypertonic saline group, 57 periods of increased ICP occurred (3.3 treatments/patient). 7.2% NaCl/HES 200/0.5 was effective in 55 episodes (96.5%), reducing ICP to <15 mmHg within 6.0 (1.2–15.0) min. In one episode, hypertonic saline (3 ml/kg) was only effective after an additional bolus of thiopental 3 mg/kg was given and, in another episode, ICP could not be reduced below 15 mmHg by an infusion of up to 3.1 ml/kg of hypertonic saline. Finally, mild hyperventilation (etCO_2 _~28–30 mmHg) achieved the target ICP value <15 mmHg.

The median dose of mannitol (145 (70–332) ml/application; 1.8 (0.45–6.5) ml/kg) required to reduce ICP below 15 mmHg was significantly higher than that of hypertonic saline (100 (35–250) ml/application; 1.4 (0.3–3.1) ml/kg). Repeated administration of mannitol caused an increase of the required single dose in six out of 15 patients (40%) and a decrease in two patients (13%). Repeated administration of hypertonic saline caused an increase of the required single dose in two patients (12%) and a decrease in seven patients (41%).

### Clinical chemistry

Hematocrit was not significantly changed by infusion of mannitol (0.3 (0.27–0.42) vs 0.29 (0.26–0.40)) and hypertonic saline (0.29 (0.24–0.37) vs 0.29 (0.24–0.36)). A temporary, but statistically significant increase of serum sodium occurred after infusion of the hypertonic saline from 143 (136–148) mmol/l to 148 (144–153) mmol/l (*p *< 0.001). Serum osmolarity increased significantly after infusion of hypertonic saline: 284 (273–300) mosm/kg to 300 (284–319) mosm/kg (*p *< 0.001), as well as after infusion of mannitol: 286 (270–315) mosm/kg to 295 (278–327) mosm/kg (*p *< 0.001).

### Outcome

Ten patients (58.8%) assigned to the group receiving hypertonic saline survived, the remaining seven patients died (41.2%). In the group with the mannitol treatment, six patients survived (40.0%) and nine patients died (60.0%). The chi-square test revealed no statistical significance.

In patients who survived, a lower dose of the osmotic agent had been administered. Survivors in the hypertonic saline group received a significant lower dose of 1.4 (0.32–2.8) ml/kg hypertonic saline. In non-survivors, the dosage given was 1.7 (0.9–3.1) ml/kg (*p *< 0.05). In the mannitol group, patients who survived received 1.7 (0.5–3.4) ml/kg mannitol versus 1.9 (1.0–6.5) ml/kg mannitol in patients who died (*p *= NS). Therefore, a statistical significance regarding the influence of the specific osmolarity, either of hypertonic saline or mannitol, given with each treatment, on changes of the cerebral hemodynamics (ICP, CPP) or patients' individual outcomes could not be analyzed.

## Discussion

The strong relationship between incidence of increased ICP and outcome in patients with neuronal damage emphasizes the vulnerability of the injured brain and the need for adequate treatment. The management of severely injured neurosurgical patients has changed over recent decades, especially regarding the introduction and acceptance of clinical guidelines among neurosurgeons and intensivists [4,10,23,24]. It has become a generally accepted treatment goal to keep the CPP above 70 mmHg, because episodes of CPP <60 mmHg or ICP >20 mmHg are associated with a worse outcome [6-8]. These goals are incorporated into current treatment protocols, which are constantly analyzed with regards to their efficacy and feasibility, and updated accordingly. Osmotic agents are important components of all treatment protocols, especially mannitol as it is a well-established treatment for increased ICP following brain injury. Surveys of the critical care management of head-injured patients show that 83% of the centers in the United States and 100% of the centers in the United Kingdom used mannitol to control ICP [25-27]. The clinical use of mannitol is, however, limited by renal complications and the fast increase of the osmotic gradient followed by its reversal due to disruption of the blood-brain barrier (BBB) [28-31]. Furthermore, mannitol (at concentrations which may be reached in clinical conditions) and the hyperosmotic stress itself can activate the process of apoptotic cell death [32].

Recent data have demonstrated different osmotic effects of mannitol. Videen and co-workers [33] observed that after administration of 1.5 g/kg bolus of mannitol in six patients with acute complete middle cerebral artery infarctions, the brain in the non-infarcted hemisphere shrank more than in the infarcted hemisphere. This may increase the inter-hemispheric pressure difference and worsen tissue shift [33].

Hypertonic saline is an interesting alternative to mannitol, because there is experimental and clinical evidence that it can reduce ICP and improve CPP [34-39]. Experimental studies in animals suffering from a combination of hemorrhagic shock and head trauma demonstrated a significant reduction of ICP, an improvement of CPP and/or a reduction of brain edema [34-36,40,41].

The efficacy of hypertonic saline after isolated brain injury, however, has rarely been investigated. Qureshi *et al*. [22] examined different concentrations of hypertonic saline (23.4%, 3.0%) versus mannitol after isolated experimental intracerebral hemorrhage in a canine model. The acute effects on ICP and CPP were most prominent after infusion of hypertonic saline 23.4%, but were better sustained after infusion of hypertonic saline 3%. The water content was highest after mannitol infusion in most regions of the brain, especially in the white matter ipsilateral to the hematoma. The authors speculated that these results were due to a certain permeability of the BBB. The most positive effect on water content was seen after hypertonic saline 3% [22].

Berger *et al*. [42] compared the efficacy of hypertonic saline and mannitol to reduce ICP after a combination of two different neuronal injuries. Initially, a cold-induced focal lesion was used to induce a vasogenic brain edema in rabbits, then intracranial hypertension was induced by a further inflation of an epidural balloon. The authors demonstrated that hypertonic solution as well as mannitol can reduce the ICP efficiently. After the first application, the effect of mannitol was enhanced compared with the hypertonic solution (98 ± 14 min vs 189 ± 27 min; *p *< 0.054), but became the same after repeated applications. It is remarkable that mannitol was more effective in decreasing the water content in brain tissue in the traumatized hemisphere, whereas hypertonic solution lowered the water content in the contralateral brain tissue. An accumulation of mannitol could occur, followed by a possible reversal of the local osmotic gradient. These different effects on brain tissue could be an explanation for the failed therapeutic efficiency after mannitol and emphasized the advantages of hypertonic solutions [42]. Furthermore, Prough *et al*. observed a higher regional cerebral blood flow in dogs with induced intracerebral hemorrhage after hypertonic saline without any increase of the CPP [43].

The positive effect of 7.2% hypertonic saline on ICP has also been demonstrated in several clinical studies investigating patients with therapy-refractory ICP increase due to isolated brain injury but without hemorrhagic shock [21,44-46]. Hypertonic saline had no effects on MAP in these euvolaemic patients [46].

Schwarz *et al*. [47] evaluated the efficacy of hypertonic saline hydroxyethyl starch 7.55% in comparison with mannitol 20% in stroke patients with increased ICP. Hypertonic saline hydroxyethyl starch was effective in all, mannitol in only 70% of patients. The maximum ICP decrease was seen 25 min after the start of hypertonic saline infusion and 45 min after the start of mannitol infusion. There was no constant effect on CPP in the hypertonic saline group, whereas CPP rose significantly in the mannitol-treated group. The authors concluded that hypertonic saline hydroxyethyl starch seems to lower ICP more effectively but does not increase CPP as much as mannitol [47].

Hypertonic saline has also been used to reduce ICP in patients with brain tumors or subarachnoid hemorrhage. Suarez *et al*. [48] reported a significant decrease of ICP and increase of CPP in these patients, when application of mannitol had been previously unsuccessful. Similar results were observed by Horn *et al*. in patients with traumatic brain injury and subarachnoidal hemorrhage, where hypertonic saline 7.5% adequately reduced ICP after mannitol therapy had failed [44].

Based on these findings, patients with isolated head trauma can also be expected to benefit from hypertonic saline. This patient population covers some specific patho-physiological conditions, characterized by diffuse axonal injuries, hemorrhages, and necrotic and edematous tissue, which can lead to different therapeutic strategies and a failed positive effect of hypertonic saline compared with patients with other intracranial mass lesions [49,50]. Munar *et al*. [51] investigated the acute effects of 7.2% hypertonic saline on ICP, cerebral blood flow and systemic hemodynamics in patients with moderate and severe traumatic brain injury during the first 72 h after injury. Hypertonic saline significantly reduces ICP without changes in MAP and relative global cerebral blood flow, expressed as 1/AVDO_2_. These results suggest that hypertonic saline decreases ICP by means of an osmotic mechanism [51].

Not all studies, however, reported positive effects of hypertonic saline on ICP, especially if hypertonic saline was infused continuously. Qureshi *et al*. analyzed the effect of continuous administration of hypertonic saline 2% or 3% in patients with head trauma. They reported a higher in-hospital mortality rate in patients receiving hypertonic solutions and described no favorable impact on the rate of necessary medical interventions during the patient's treatment in the ICU. The influence of hypertonic saline on the supposedly disrupted BBB after head injury was mainly used to explain the failed effect. A disrupted BBB can lead to an accumulation of sodium resulting in an reversal of the osmotic gradient with concomitant increase of ICP [52]. However, Hartl *et al*. demonstrated a reduced water content in areas with a disturbed BBB in a model with or without a focal cryogenic brain lesion and hemorrhagic shock [53].

Our results showed that bolus application of either study medication, mannitol 15% or hypertonic saline 7.2%, significantly decreases ICP and increases CPP (Table 2). The effect of hypertonic saline on ICP was significantly better than that of mannitol. Clinically important effects of both drugs on MAP could not be determined, although some statistically significant differences were observed at a few measurement points. Therefore, it can be concluded that local cerebral dehydration is the main mechanism of both substances in decreasing ICP and optimizing CPP. The higher potency of hypertonic saline suggests that its local effect is more clearly pronounced.

However, the mechanisms whereby hypertonic solutions reduce ICP are multifactorial and are still discussed with some controversy. The main principle seems to be the 'local dehydration' of brain tissue drawing water from parenchyma to the intravascular space following an osmotic gradient [54]. Comparing this with the osmotic effect of mannitol, a second mechanism to explain the effect of the ICP-reduction must exist. This hypothesis is supported by the results of Berger *et al*. [42]. He found, in rats with induced head injury, a similar positive effect on ICP with regards to the amount and duration of the decrease, but a higher CPP in the rats receiving mannitol. Contrary to our results, the MAP increased after hypertonic saline, whereas the MAP temporarily decreased after mannitol. The authors hypothesized that the different effects of the two solutions are the result of a selective permeability of the BBB and/or the different reflection coefficients. A disrupted BBB would have to be the result of an accumulation of both solutions in the brain tissue. Therefore different mechanisms of local cerebral dehydration must exist [42]. These hypotheses are supported by the results of Worthley *et al*. and Kaufmann *et al*. Both working groups demonstrated that the ICP-decreasing effect is limited after repeated bolus applications of mannitol, but a further application of hypertonic saline lead to a further ICP reduction [55,56]. However, a direct vasodilatation of pial vessels [57-59], the reduction of blood viscosity due to enhancement of the intravascular volume, the rapid absorption of cerebrospinal fluid and restoration of the normal membrane potentials are other effects to positively affect the ICP [60,61]. Our results only support the hypothesis about the local dehydration of brain tissue. Systemic hemodynamic effects for the given dosage couldn't be demonstrated, but the decreased ICP leads to the improved CPP. All homeostatic side effects after hypertonic saline, for instance hypernatriemia and increased serum osmolarity, are temporary and without systemic hemodynamic side effects. Such complications as described in the literature, emphasize cardiac failure with lung edema, metabolic acidosis, coagulopathia subdural hematoma and central pontine myolysis as the most important [22,40,48]. With the intention of limiting the side effects of changes in electrolytes and osmolarity, a standardized laboratory measurement procedure is needed.

The substantial difference in the design of the present and a comparable study is the fact that we did not administer a fixed total dose, but infused the study medication at a defined infusion rate until ICP decreased to <15 mmHg, the primary goal of our treatment. No clinical study has so far identified an exact dose-effect relationship for hypertonic saline. Only one comparable clinical study confirms the superiority of 2.0 ml/kg of hypertonic saline 7.5% over mannitol 20% in head-injured patients [21]. This study concluded that 2 ml/kg of 7.2% NaCl/HES 200/0.5 can be recommended as an effective dose to reduce increased ICP [21]. In our study, an average dose of 1.5 ± 0.6 ml/kg of hypertonic saline adequately reduced ICP below 15 mmHg. Furthermore, because of our application mode with an defined application rate and a target ICP of <15 mmHg we could demonstrate a failed influence of the osmotic load given with each treatment.

Regardless of all positive effects in our study, there are some limitations that need to be discussed, most of all, the small patient population of each group and the heterogeneity in the underlying neurological illness. The primary intention of our study was pragmatic and adjusted on the typical clinical routine. However, we included neurosurgical patients with severe neuronal damage independent from the individual pathogenesis. To compensate for this to a certain degree, we used a randomized study design. Furthermore, until now there have been only limited data available for comparison of these two osmotic agents in a clinical setting. A small amount of evidence is available that hypertonic saline has some advantages compared with mannitol in the treatment of patients with intracranial hypertension after trauma, subarachnoid bleeding or stroke [21,47,62,63].

## Conclusion

7.2% NaCl/HES 200/0.5 and mannitol 15% are effective and safe drugs in the treatment of increased ICP, although 7.2% NaCl/HES 200/0.5 is more effective than mannitol. A dose of 1.4 ml/kg can be recommended as an initial dose. The advantage of hypertonic saline can be explained by individual local osmotic effects, because no relevant systemic changes occur. The observed effects on electrolytes and plasma osmolarity are not significantly different between the two osmotic drugs and have no clinical relevance here. Further experimental and clinical research is required to evaluate the optimal administration regime, the best treatment strategies adapted to the individual patient's needs and the impact on patients' morbidity and mortality.

## Key messages

• 	7.2% NaCl/HES 200/0.5 is more effective than mannitol in the treatment of increased ICP

• 	A dose of 1.4 ml/kg 7.2% NaCl/HES 200/0.5 can be recommended as an initial dose

• 	The local dehydration of brain tissue after application of 7.2% NaCl/HES 200/0.5 seems to be the primary mechanism for the improved CPP

## Abbreviations

BBB = blood-brain barrier; CPP = cerebral perfusion pressure; GCS = Glasgow Coma Score; ICH = intracerebral hemorrhage; ICU = intensive care unit; SAH = subarachnoid hemorrhage; SAPS = simplified acute physiology score; SHT = severe head trauma; SpO_2 _= peripheral oxygen saturation

## Competing interests

The author(s) declare that they have no competing interests.

## Authors' contributions

All of the authors were involved in designing the study and collecting data. JS and LH were involved in the statistical analysis. SG revised the article and was responsible for translation into English. All authors read and approved the final manuscript.

## Figures and Tables

**Table 1:** Demographic data of analyzed patients

	Mannitol 15% (*n *= 15)	7.2% NaCl/HES 200/0.5 (*n *= 17)
Age	47 ± 16	47 ± 16
Weight	89 ± 27	87 ± 24
Gender, M/F	8/7	9/8
Initial GCS	5.8 ± 1.4	6 ± 1.3
SAPS score	42.5 ± 13	39.6 ± 9.6
Days on ICU	23.3 ± 14.8	22.8 ± 15.5
Basic illness		
SAH	5	4
Brain infarct	4	3
Isolated SHT III°	4	6
ICH	1	3
Other	1	1
Surgical intervention	13	13

7.2% NaCl/HES 200/0.5, 7.2% hypertonic saline hydroxyethyl starch 200/0.5; GCS, Glasgow Coma Score; ICH, intracerebral hemorrhage; ICU, intensive care unit; SAPS, simplified acute physiology score; SHT, severe head trauma.

**Table 2:** Time course of heart rate, MAP, ICP and the CPP for the two different treatment groups

	Start infusion	Terminating infusion	+10 min	+30 min	+60 min
Heart rate, l/min					
7.2% NaCl/HES 200/0.5	76 [52–92]	78 [60–104]	77 [62–107]	78 [62–101]	79 [61–99]
Mannitol 15%	78 [58–95]	80 [58–96]	80 [60–95]	81 [58–93]	79 [56–96]
					
MAP, mmHg					
7.2% NaCl/HES 200/0.5	84 [64–98]	84* [68–96]	84* [67–97]	85* [74–100]	84 [63–94]
Mannitol 15%	84 [68–92]	85 [65–98]	83 [69–105]	81 [69–106]	82 [68–108]
					
ICP, mmHg					
7.2% NaCl/HES 200/0.5	22 [19–31]	15** [8–18]	12** [2–16]	10**,^++ ^[6–14]	11**,^+ ^[5–18]
Mannitol 15%	23 [19–30]	14** [7–20]	13** [4–19]	12** [6–19]	14** [7–20]
					
CPP, mmHg					
7.2% NaCl/HES 200/0.5	60 [39–78]	72** [54–85]	72** [55–89]	75**, ^#^[62–86]	73**, ^#^[58–88]
Mannitol 15%	61 [47–71]	70** [50–79]	70** [56–92]	72** [60–93]	69** [56–89]

*p < 0.05, **p < 0.0001 compared with start infusion. +*p *< 0.0001, ++*p *< 0.01, ^#^*p *< 0.05 between treatment regimes. HR, heart rate; CPP, cerebral perfusion pressure; ICP, intracranial pressure; MAP, mean arterial pressure.
